# Effects of RhoC downregulation on the angiogenesis characteristics of myeloma vascular endothelial cells

**DOI:** 10.1002/cam4.2208

**Published:** 2019-05-07

**Authors:** Zhihua Zhao, Kai Liu, Xiangyu Tian, Miaomiao Sun, Na Wei, Xiaoyan Zhu, Hongmei Yang, Tong Wang, Guozhong Jiang, Kuisheng Chen

**Affiliations:** ^1^ Henan Province Key Laboratory of Tumor Pathology Department of Pathology of The First Affiliated Hospital of Zhengzhou University Zhengzhou People’s Republic of China; ^2^ Zhengzhou Central Hospital Affiliated to Zhengzhou University Zhengzhou Henan People’s Republic of China; ^3^ Department of Pathology of The First Affiliated Hospital of Zhengzhou University Zhengzhou Henan People’s Republic of China; ^4^ The Affiliated Tumor Hospital of Zhengzhou University Zhengzhou Henan People’s Republic of China; ^5^ Department of Histology and Embryology, School of Basic Medical Sciences Zhengzhou University Zhengzhou Henan People’s Republic of China; ^6^ Henan Province Medical College Zhengzhou Henan People’s Republic of China

**Keywords:** angiogenesis, lentiviral vector, myeloma, RhoC, vascular endothelial cells

## Abstract

**Background:**

Tumor angiogenesis plays an important role in disease progression, and RhoC has been previously found to be expressed in vascular endothelial cells (VECs); however, its role in tumor angiogenesis requires clarification. This study aimed to explore the effects of RhoC downregulation on the cytoskeleton, pseudopod formation, migration ability, and canalization capacity of myeloma vascular endothelial cells (MVECs) in vitro.

**Materials and methods:**

The expression of RhoC in MVECs and human umbilical vein endothelial cells (HUVECs) was knocked down by shRNA, and the expression levels of RhoC mRNA were detected by quantitative reverse transcription polymerase chain reaction (qRT‐PCR). The cytoskeletal changes and pseudopods were observed by laser scanning confocal and scanning electron microscopy; VECs were incubated in two‐dimensional Matrigel and three‐dimensional microcarriers to observe tube‐like structures and budding status, respectively. The protein expression of RhoC, phosphorylation of mitogen‐activated protein kinase (p‐MAPK), and Rho‐associated coiled‐coil kinase (ROCK) was determined by Western blotting. The expression of RhoC in VECs was downregulated by RhoC shRNA, thereby decreasing the number of pseudopods, two‐dimensional tube‐like structures, and buds.

**Results:**

When RhoC was downregulated, the expression levels of ROCK and phosphorylation of MAPK were both decreased (*P* < 0.05). Moreover, the expression levels of RhoC and phosphorylation of MAPK and three‐dimensional budding numbers were higher in MVECs than in HUVECs (*P* < 0.05). The downregulation of RhoC expression in MVECs and HUVECs inhibited pseudopod formation, migration, canalization ability, and angiogenesis (*P* < 0.05).

**Conclusion:**

Our data indicated that MVECs and HUVECs were well suited for angiogenesis research, but the former cell type was shown to be more advantageous in terms of budding numbers. RhoC plays a pivotal role in MVECs angiogenesis, and the downregulation of RhoC expression could inhibit angiogenesis via the RhoC/MAPK and RhoC/ROCK signaling pathways.

## 1 INTRODUCTION

RhoC is a member of the Ras‐homologous (Rho) GTPase family, which comprises important signaling molecules that are involved in regulating processes associated with dynamic changes in the cytoskeleton, such as cell migration and proliferation.^1^, ^2^ As shown previously in a study of esophageal carcinomas, RhoC protein expression can upregulate vascular endothelial growth factor (VEGF), which is closely associated with tumor angiogenesis.^3^

Angiogenesis is initiated and regulated by many factors and is an extremely complex process that is mediated by a variety of inducing factors and includes multiple steps, such as vascular endothelial cell proliferation and migration, extracellular matrix (ECM) degradation and remodeling, and vascular formation.^4^, ^5^ Angiogenesis is required for tumor growth and metastasis.^6^, ^7^ RhoC expression is related to cell proliferation, migration, and cytoskeletal alterations.^8^, ^9^ However, RhoC was also found to be expressed in vascular endothelial cells (VECs);^3^ therefore, its expression might be associated with the angiogenesis of VECs.

In angiogenesis studies, human umbilical vein endothelial cells (HUVECs) are usually selected as the model cell line.^10^, ^11^ Normal bone marrow plasma cells expressed more proangiogenic genes than antiangiogenic genes and induced angiogenesis in vitro. The accumulation of plasma cells can induce basal vascularization at the bone marrow level.^12^ Myeloma angiogenesis is regulated by various factors, such as the bone marrow microenvironment and hypoxia.^13^, ^14^ Given the substantial differences in structure and conformation between normal HUVECs and myeloma vascular endothelial cells (MVECs), HUVECs, and MVECs were selected for this study. By knocking down RhoC, we investigated the associations between MVECs and angiogenesis as well as the possible mechanisms through which RhoC affects vascular formation from endothelial cells, uncovering novel mechanisms associated with angiogenesis and providing new therapeutic strategies for targeting tumor angiogenesis.

## 2 MATERIALS AND METHODS

### 2.1 Reagents

The shRNA lentiviral vectors that were used to knockdown of RhoC expression were purchased from GenePharma (Suzhou, China). An anti‐RhoC antibody was purchased from Abcam (Cambridge, MA, USA). The anti‐mitogen‐activated protein kinase (MAPK) and anti‐Rho‐associated coiled‐coil kinase (ROCK) antibodies were purchased from Proteintech (Chicago, IL, USA). Phalloidin was purchased from Cytoskeleton (Denver, CO, USA). DAPI was purchased from Santa Cruz Biotechnology (Dallas, Texas, USA). Ezol was purchased from GenePharma; Cytodex3 was purchased from GE Healthcare (Uppsala, Sweden), and HRP‐conjugated secondary antibody was purchased from Jackson (West Grove, PA, USA).

### 2.2 Cells

MVECs and HUVECs were purchased from Boquaner Biotech Ltd (Shanghai, China).

### 2.3 Lentiviral transduction of VECs

*Two* types of VECs (MVECs and HUVECs) were investigated in this study, and each type was grouped into a negative control group (NC group) and an experimental group (S group). After trypsin digestion, cells were added to a 24‐well culture plate at a concentration of 5 × 10^4^ cells per well, which was followed by the addition of 500 μL of 10% fetal bovine serum/Dulbecco's modified Eagle's medium (DMEM) culture medium (to each well); culture plates were placed in an incubator at 37°C for incubation. On the next day, a lentiviral stock solution was diluted with cell culture medium at a ratio of 1:10 (v/v, stock solution: culture medium), and the culture medium was removed from each well and replaced with 500 μL of the diluted lentiviral solution. After a 12h incubation, complete culture solution was added to each well to replace the old culture medium and incubated for 48 hours. Subsequently, transfection efficiency was monitored, during which five 200× visual fields were randomly selected, and 100 cells were counted; the infection rate was defined as the average percentage of green fluorescent cells relative to the total number of observed cells.

### 2.4 Detection of RhoC mRNA expression after lentiviral transfection by quantitative reverse transcription polymerase chain reaction

The forward and reverse primers, respectively, for the target gene *RhoC* were 5′‐CAGTGCCTTTGGCTACCTTG‐3′ and 5′‐CCCTCCGACGCTTGTTCTT‐3′, and those for *GAPDH* were 5′‐CATGAGAAGTATGACAACAGCCT‐3′ and 5′‐AGTCCTTCCACGATACCAAAGT‐3′ (Table 1). After lentiviral transfection, the cells were incubated in a 6‐well culture plate until each well reached confluence. This step was followed by the addition of 300 μL Ezol to lyse the cells to extract total RNA. With three duplicate wells for each group, RNA samples were subjected to reverse transcription and PCR amplification. The PCR procedure was carried out to include pre‐denaturation at 95°C for 3 minutes, denaturation at 95°C for 3 seconds, annealing at 62°C for 40 seconds, and extension at 72°C for 5 minutes. In total, 40 amplification cycles were conducted, and the amplified fragment had a size of 113 bp. The measured cycle threshold values were used to calculate the 2^−ΔΔCt^ value to compare the relative quantitative expression of mRNA among the groups, and each group was measured in triplicate.^15^

**Table 1:** Primers for RhoC and GAPDH

Primers	Sense (5′‐3′)	Antisense (5′‐3′)
RhoC	CAGTGCCTTTGGCTACCTTG	CCCTCCGACGCTTGTTCTT
GAPDH	CATGAGAAGTATGACAACAGCCT	AGTCCTTCCACGATACCAAAGT

### 2.5 Pseudopod observation

The NC group and the S group of cells were separately incubated on cover glass. After reaching 60% confluence, the cells were fixed with paraformaldehyde for 20 minutes and treated with Triton‐100x for 20 minutes. A 5‐μL portion of phalloidin labeled with rhodamine was diluted in 200 μL phosphate‐buffered saline (PBS) solution, and the resulting mixture was applied to the aforementioned cover glass; cells samples were incubated for 30‐60 minutes, followed by a 10min incubation with DAPI. The cells were observed using a laser scanning confocal microscope (Nikon Eclipse Ti, Japan); images were analyzed using Photoshop to magnify (400×) the observed images.

### 2.6 Scanning electron microscopy for the observation of cellular pseudopods and cytoskeletons

When the NC group and the S group of cells grew to 60% confluency on the cover glass, they were rinsed with PBS, fixed with 3% glutaraldehyde (precooled to 4°C) and sat overnight at 4°C. Next, the cells were rinsed with PBS twice, each time for 10 minutes, and fixed with osmic acid (precooled to 4°C) for 1 hour at 4°C. This step was followed by a gradient alcohol dehydration process (15 minutes each time), a freeze‐drying process, and a vacuum gold‐sputtering process. The prepared samples were observed under a scanning electron microscope (Phenom‐World, Netherlands), and images were obtained using visual fields magnified 5000×.

### 2.7 Scratch test

Each group of cells was inoculated into a 24‐well culture plate at a concentration of 1 × 10^4^ cells/well, and three replicate wells were set up for each group. At confluence, the cells in each group were scratched and subjected to serum‐free incubation for 24 hours. The cells were then observed and imaged in a visual field magnified 100× using an inverted microscope. ImageJ software was used to analyze the rate at which the scratched areas were filled.

### 2.8 Two‐dimensional canalization on Matrigel

Matrigel was thawed at 4°C, and in the meantime, a 24‐well culture plate and 200‐μL tips were precooled. Matrigel was mixed with serum‐free medium at a ratio of 1:1, and the resulting mixture was added to the 24‐well culture plate at a concentration of 200 μL per well. The plate was then placed in an incubator at 37°C for 1 hour to allow the mixture to solidify into a gel. Each group of cells was trypsin‐digested until a density of 1 × 10^5^ cells/mL was reached. Next, 1 mL of the resulting cell suspension was added to each well, and the plate was placed in an incubator at 37°C for 24 hours, during which the canalization status was monitored. The canalization status was assessed by the following equation: node number × branch number, as observed under an inverted microscope. Five visual fields at a magnification of 100× were randomly selected for each group to calculate the average value.

### 2.9 Three‐dimensional budding on the Cytodex3 microcarrier

One hundred milligrams of Cytodex3 was subjected to sterilization treatment, according to the product manual, and rinsed once with lukewarm culture medium. This solution was then added to 2 mL DMEM to obtain the diluted solution (50 mg/mL). Each group of transfected cells was digested and resuspended to reach a density of 2 × 10^6^ cells/mL. A 1‐mL portion of the resulting suspension was added to an Eppendorf tube, to which 50 μL of Cytodex3 was then added for mixing. Matrigel was spread on the well of a 24‐well plate. Microcarriers that were fully covered with cells were then selected, rinsed with prewarmed culture medium, and centrifuged at a low speed to remove the cells in the microcarrier suspension. After the supernatant was carefully removed, the mixture remaining in the centrifuge tube was homogeneously mixed with Matrigel that contained 10 ng/mL VEGF, and the resulting mixture was added to the abovementioned 24‐well culture plate, which had been loaded with the gel. The plate was then placed in an incubator at 37°C to allow the gel‐containing mixture to solidify. Next, new culture medium was added to the 24‐well culture plate, which was then placed in the incubator for incubation; budding status was monitored consecutively for 4 days. Effective budding length was defined as the diameter of the ball, and the budding number for 10 microcarriers was measured in triplicate from a visual field at 200× magnification for each group of cells; the average values were calculated based on triplicate measurements.

### 2.10 Protein levels of Phosphorylation of MARK, ROCK, and RhoC

The cells in the NC and S groups were all lysed for the extraction of proteins. After boiling and denaturing, the protein samples were introduced to a culture plate at a dose of 10 μL per well. Electrophoresis (Bio‐Rad Laboratories, Inc, Hercules, CA, USA) was conducted at 80 V for 30 minutes and then 120 V for 60 minutes. Next, the samples were transferred to a polyvinylidene difluoride membrane (EMD Millipore, Billerica, MA, USA) by applying an 80 V transmembrane voltage for 90 minutes, followed by overnight incubation with *RhoC* (1 μg/mL, ab64659; Abcam) and incubation with *ROCK* (1:500, 21850‐1‐AP) and Phosphorylation of *MAPK* (1:500, 66234‐1‐Ig) (both from Proteintech) primary antibodies at 4°C; then, a final incubation with HRP‐conjugated secondary antibody (1:5000, 115‐035‐003; Jackson) was performed on the next day. The protein expression in each group of cells was measured in triplicate based on electrochemiluminescence; ImageJ software was used for quantitative grayscale analysis.

### 2.11 Statistical analysis

The statistical software SPSS 17.0 (SPSS, Inc, Chicago, IL, USA) was used to test the normality of the data in this study. Data conforming to a normal distribution were expressed as $\overset{¯}{x}$ ± SD, and a *t*‐test was used to compare the two samples (*P* < 0.05).

## 3 RESULTS

### 3.1 Effect of RhoC gene silencing

**Figure 1:**
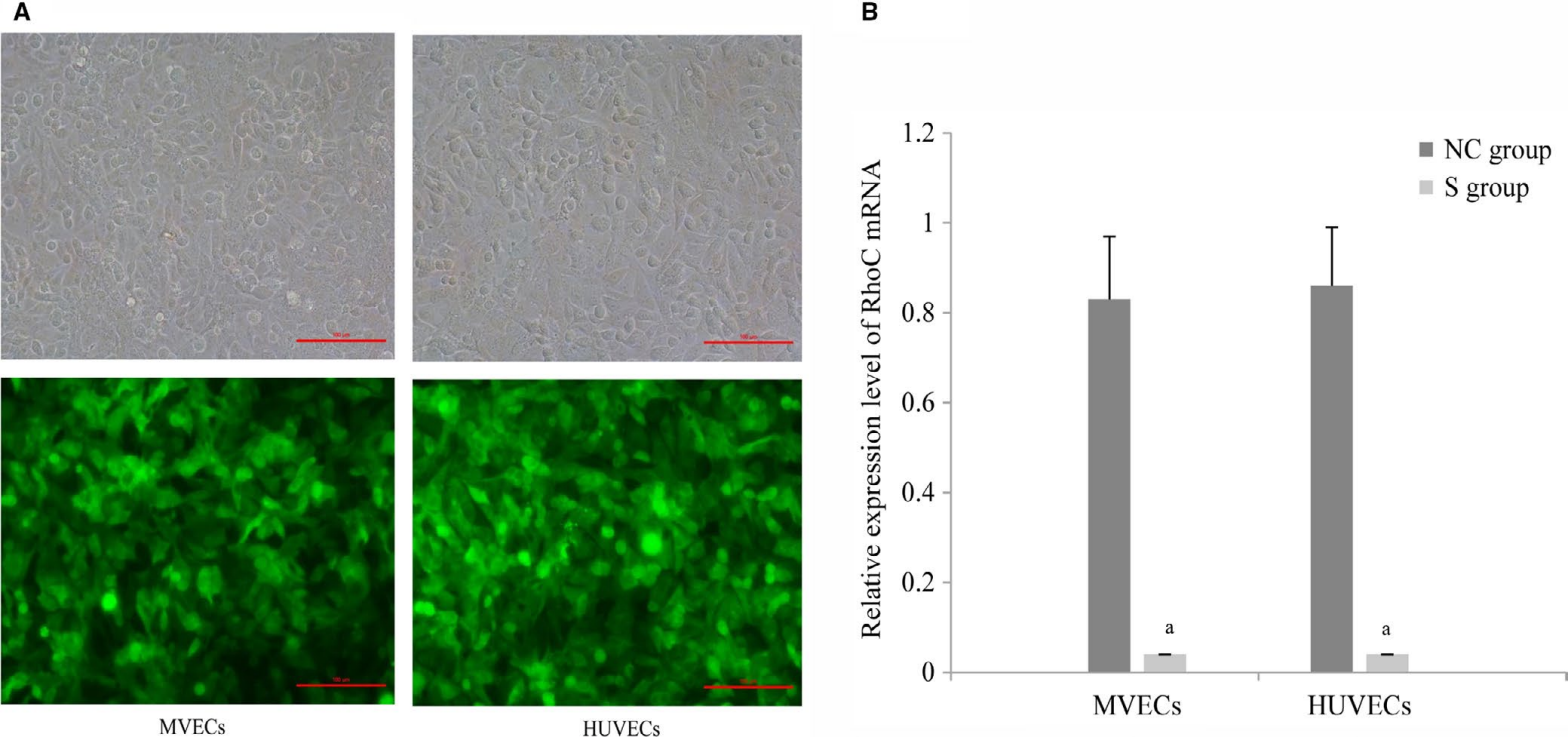
Observational evaluation of lentiviral transfection. (A) in vascular endothelial cells by fluorescence microscopy (200×, scale = 100 μm). MVECs and HUVECs were transduced with lentiviral vectors encoding RhoC‐targeting shRNA. Expression of *RhoC* mRNA (B) in each group of VECs (n = 3). MVECs and HUVECs were transduced with lentiviral vectors encoding RhoC‐targeting shRNA, and mRNA expression was determined by qRT‐PCR. (^a^, *P* < 0.05, compared to the NC group). HUVECs, human umbilical vein endothelial cells; MVECs, myeloma vascular endothelial cells; NC, negative control; VECs, vascular endothelial cells

Inverted fluorescence microscopy revealed that both types of cells transfected with lentiviruses expressed green fluorescent protein, and the transfection efficiency was greater than 80% (Figure 1A). Quantitative reverse transcription polymerase chain reaction (qRT‐PCR) revealed significantly lower *RhoC* mRNA levels in the S group than in the NC group for both MVECs (*t* = 9.50, *P* < 0.05) and HUVECs (*t* = 10.92, *P* < 0.05) (Figure 1B).

### 3.2 Observation of pseudopods

**Figure 2:**
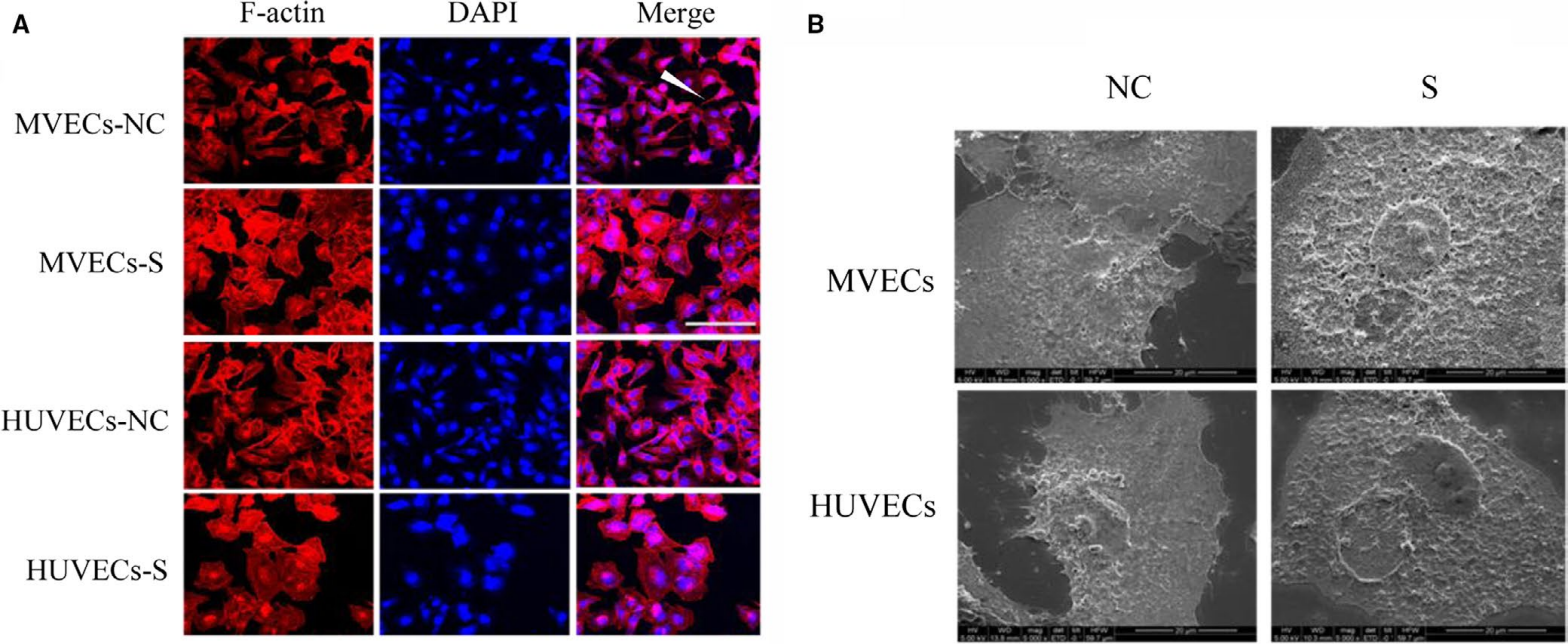
Observation of pseudopods. (A) in each group of vascular endothelial cells, as observed by laser scanning confocal microscopy (400×, scale = 50 μm). The groups were as follows: MVECs‐NC, myeloma vascular endothelial cells transfected with the negative control, and MVECs‐S, MVECs transfected with the RhoC shRNA vector. HUVECs‐NC, human umbilical vein endothelial cells transfected with the negative control; HUVECs‐S, HUVECs transfected with the RhoC shRNA vector. Pseudopods (B) in VECs with or without RhoC knockdown, as observed by scanning electron microscopy (5000×, scale = 20 μm). HUVECs, human umbilical vein endothelial cells; MVECs, myeloma vascular endothelial cells; NC, negative control; VECs, vascular endothelial cells

For MVECs, the number of pseudopods in the *RhoC* knockdown group (S group) was less than that in the NC group (*t* = 10.92,* P* < 0.05), with the cytoskeleton of cells in the S group exhibiting polygonal shapes. Similar results were observed in the HUVECs (*t* = 21.17, *P* < 0.05) (Figure 2A,2 & Table 2).

**Table 2:** Percentage of pseudopodia formation in each group

Groups	NC group (mean ± SD)	S group (mean ± SD)	T value	*P* value
MVECs	0.87 ± 0.01	0.12 ± 0.06^a^	69.22	0.00
HUVECs	0.94 ± 0.02	0.07 ± 0.01^a^	21.17	0.00

Abbreviations: HUVECs, human umbilical vein endothelial cells; MVECs, myeloma vascular endothelial cells; NC, negative control; S, cells transduced with the RhoC shRNA vector.

a *P* < 0.05, compared to the NC group.

### 3.3 Vascular endothelial cell migration and movement

**Figure 3:**
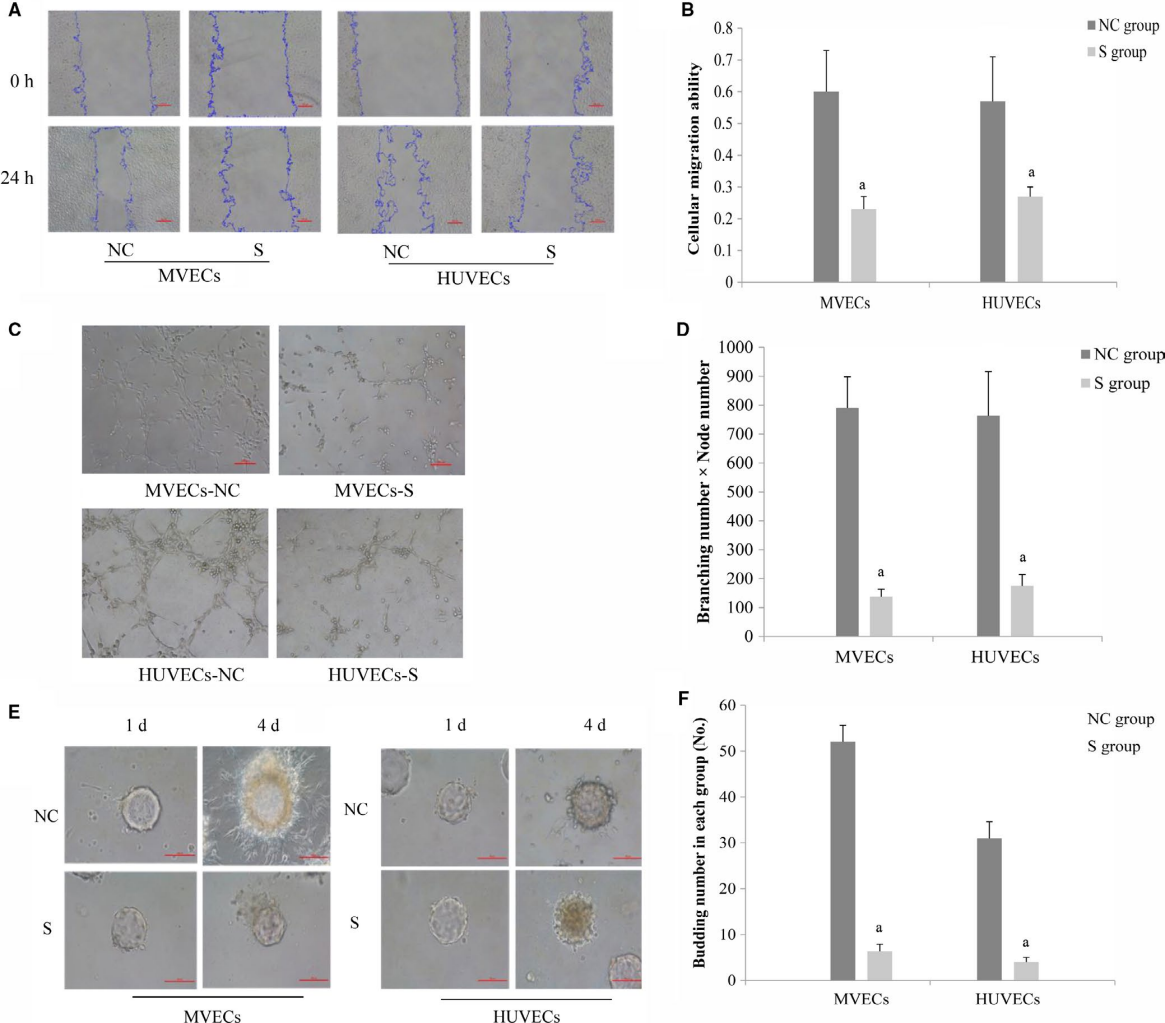
The effect of RhoC on cellular migration/canalization/budding ability. (A) in vascular endothelial cells (100×, scale = 100 μm). After RhoC knockdown, migration was assessed by a scratch assay. Analysis of scratch healing ability (B) in each group of VECs (n = 3). Effect of RhoC on canalization (C) in each group of VECs grown on Matrigel (100×, scale = 100 μm). Quantification of canalization (D) in each group of VECs grown on Matrigel (n = 5). Budding (E) in each group of VECs grown on microcarrier beads with and without RhoC knockdown (n = 5, 200×, scale = 100 μm). Analysis of budding (F) in each group of vascular endothelial cells grown on microcarrier beads (^a^, *P* < 0.05, compared to the NC group). NC, negative control; VECs, vascular endothelial cells

MVECs in the S group had a significantly slower migration speed than their counterparts in the NC group (*t* = 4.48, *P* < 0.05). Similar results were obtained for HUVECs (*t* = 3.73, *P* < 0.05). There was no significant difference in migration speed between the two types of cells in the NC group (*t* = 0.21, *P* > 0.05), as shown in Figure 3A,3.

### 3.4 Canalization of VECs

After incubation for 12 hours on Matrigel, the MVECs in the S group showed significantly decreased canalization compared to that in the NC group (*t* = 13.25, *P* < 0.05); similar results were observed for HUVECs as well (*t* = 8.36, *P* < 0.05). In contrast, there was no significant difference in canalization between the MVECs and HUVECs in the respective NC groups (*t* = 0.33, *P* > 0.05; Figure 3C,3).

### 3.5 Vascular endothelial cell budding

After incubation on microcarrier beads for 1 day and 4 days, both MVECs (*t* = 20.20, *P* < 0.05) and HUVECs (*t* = 12.50, *P* < 0.05) in the S group exhibited significantly decreased budding compared to that of the MVECs and HUVECs in the NC group; MVECs in the NC group demonstrated significantly enhanced budding compared to the HUVECs in the NC group (*t* = 7.13, *P* < 0.05), as shown in Figure 3E,3.

### 3.6 Western blot analysis of phosphorylation of MAPK, ROCK, and RhoC protein levels

**Figure 4:**
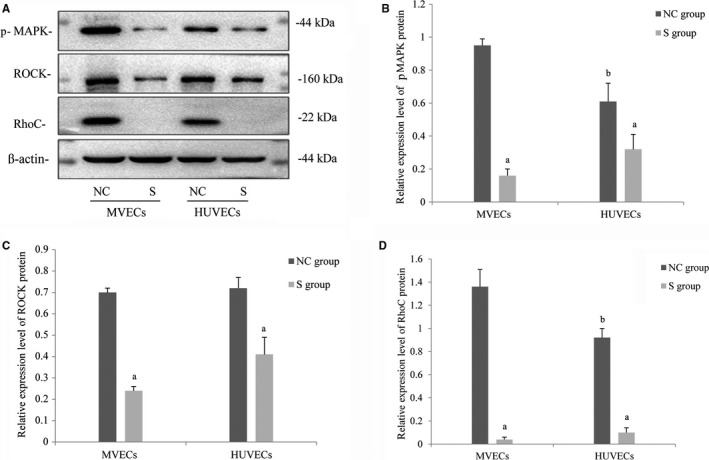
Protein expression of vascular endothelial cells. (A) Western blot analysis of protein expression in each group of VECs. (B) Western blot analysis for the quantification of RhoC expression in each group of VECs. (C) Western blot analysis for the quantification of phosphorylated MAPK protein expression in each group of VECs. (D) Western blot analysis for the quantification of ROCK protein expression in each group of VECs (^a^, *P* < 0.05, compared to the NC group) (^b^, *P* < 0.05, compared to MVECs in the NC group). MAPK, mitogen‐activated protein kinase; MVECs, myeloma vascular endothelial cells; NC, negative control; ROCK, Rho‐associated coiled‐coil kinase; VECs, vascular endothelial cells

As shown in Figure 4, phosphorylation of MAPK protein expression in the S group of MVECs was significantly lower than that in the NC group of MVECs (*t* = 25.16, *P* < 0.05), and the same effect was observed for HUVECs (*t* = 3.63, *P* < 0.05), as shown in Figure 4A,4. ROCK protein expression in the S group was significantly lower than that in the NC group for both MVECs (*t* = 28.17, *P* < 0.05) and HUVECs (*t* = 5.88, *P* < 0.05), as shown in Figure 4C. RhoC protein expression in the S group of MVECs was significantly lower (*t* = 15.58, *P* < 0.05) than that in the NC group; similar results were obtained for HUVECs (*t* = 16.22, *P* < 0.05), as shown in Figure 4D.

## 4 DISCUSSION

RhoC is a member of the Rho GTPase family and belongs to a class of small molecular G proteins. RhoC protein expression in endothelial cells is intimately associated with tumor angiogenesis.^3^ Angiogenesis involves endothelial cell pseudopods, migration and movement, canalization, budding, and vascularization.

After transfecting VECs with RhoC shRNA in this study, the level of RhoC mRNA in the experimental group of cells decreased by 95% compared to expression in the control group. Therefore, RhoC expression was knocked down to explore the relationship between RhoC and angiogenesis using endothelial cells.

Moreover, a prerequisite for cellular migration is the formation of lamellipodia, filopodia, and focal adhesion.^16^ Laser scanning confocal and scanning electron microscopy were used in this study to observe pseudopod conformation, showing that after RhoC knockdown, the number of filopodia markedly decreased, the cellular conformation was more regular, and the width of lamellipodia was restricted. This phenomenon is consistent with the result obtained by Ridley^17^ who showed that RhoC could restrict the width of cell pseudopods.

Research on the Rho family has mainly focused on the cytoskeleton, cellular mobility, cell proliferation, and tumor cell infiltration and metastasis.^18^ In recent years, an increasing number of studies have found that RhoC is not only involved in tumor invasion and metastasis^18^, ^19^ but also plays an important role in tumor development and progression. Studies on hepatocellular carcinoma (HCC) have suggested that RhoC promotes the evolution of healthy hepatocytes into malignant cells by promoting cell migration;^20^ this indicates that RhoC may be a new oncogene for HCC. By investigating of RhoC, it has been found that its expression in tumors, such as esophageal squamous cell carcinoma^3^ and HCC,^21^ is intimately associated with tumor angiogenesis.

For the scratch test, RhoC downregulation could inhibit endothelial cell migration. The regulation of cellular migration by Rho GTPase is achieved by affecting the activities of actin and myosin as well as cell adhesion. ROCK can increase myosin phosphorylation such that it regulates actin contractility. To verify the effects of RhoC in the involvement of VECs in angiogenesis, VECs were cultured on a Matrigel‐covered two‐dimensional culture plate and on a three‐dimensional microcarrier. The results showed that the two‐dimensional tube‐like structures of the two types of VECs were markedly decreased by RhoC downregulation, with a reduction in budding observed on the three‐dimensional microcarrier. Therefore, it was confirmed that RhoC plays an important role in angiogenesis in VECs. The results of the expression of RhoC and ROCK proteins shown by Western blot showed that RhoC silencing decreased ROCK protein expression, which is consistent with the finding of Rong et al^22^ who showed that the downstream effector molecule of RhoC is ROCK. Some studies have shown that RhoC activates ROCK by binding with ROCK and then phosphorylates the myosin light chain, participates in cell aggregation and fiber contraction, and promotes cell metastasis and infiltration.^23^

The MAPK signaling pathway exists widely in cells and is involved in many biological processes, such as cell growth, development, division, differentiation, and apoptosis. This pathway is also closely associated with the development and progression of multiple malignant tumors. Regarding the MAPK family members, increased activation of extracellular regulated protein kinase (ERK) can stimulate angiogenesis. The ERK signaling pathway plays a pivotal role in malignant processes, such as the development and proliferation of tumors and tumor angiogenesis.^10^ One study on prostate cancer showed that the RhoC/ROCK signaling pathway could upregulate the phosphorylation of MAPK, and the phosphorylation level of MAPK in prostate cancer tissues was significantly higher than that in adjacent tissues and was related to tumor cell metastasis.^24^ Therefore, with respect to the mechanisms through which VECs participate in angiogenesis, we focused on detecting phosphorylation of MAPK protein expression and found that the downregulation of RhoC interfered with phosphorylation of MAPK expression. Based on these findings, we suggest that the RhoC/MAPK signaling pathway plays a pivotal role in angiogenesis.

Research on breast cancer has reported that interfering with *RhoC* gene expression could inhibit the proliferation and infiltration abilities of tumor cells through a mechanism that likely involves the simultaneous downregulation of matrix metalloproteinase‐9 (MMP‐9).^25^ The ECM primarily exists between cells, and therefore, angiogenesis requires the degradation of the ECM. Notably, the most important enzyme for ECM degradation is MMP‐9. Therefore, the reduction in angiogenesis by the downregulation of RhoC may be associated with inhibition of MMP‐9 expression. The above speculation, once confirmed, would validate a report by Shuli et al,^20^ which showed that overexpression of RhoC stimulates MMP‐9 expression to promote angiogenesis. However, whether RhoC/MMP‐9 is involved in angiogenesis requires further investigation.

In conclusion, as confirmed by in vitro experimental detection, RhoC plays a pivotal role in MVECs angiogenesis, and the downregulation of RhoC expression could inhibit angiogenesis via the RhoC/MAPK and RhoC/ROCK signaling pathways. Moreover, MVECs were superior to HUVECs when used in three‐dimensional systems for the observation of budding.

## ETHICS APPROVAL AND CONSENT TO PARTICIPATE

All experimental procedures involving human samples were approved by the Life Science Ethics Review Committee of Zhengzhou University.

## CONFLICT OF INTEREST

The authors declare that they have no competing interests.

## AUTHORS' CONTRIBUTIONS

KC and MS conceived and designed the experiments; XT, KL, NW, ZW, and TW performed the experiments; GJ, ZZ, XZ, and LD analyzed all the data; and XT, KL, MS, and HY wrote the manuscript.

## Data Availability

The datasets generated in this study are available from the corresponding author on reasonable request.
